# A genomic perspective on the potential of termite-associated *Cellulosimicrobium cellulans* MP1 as producer of plant biomass-acting enzymes and exopolysaccharides

**DOI:** 10.7717/peerj.11839

**Published:** 2021-07-28

**Authors:** Nguyen Thi-Hanh Vu, Tung Ngoc Quach, Xuan Thi-Thanh Dao, Ha Thanh Le, Chi Phuong Le, Lam Tung Nguyen, Lam Tung Le, Cuong Cao Ngo, Ha Hoang, Ha Hoang Chu, Quyet-Tien Phi

**Affiliations:** 1 Institute of Biotechnology, Vietnam Academy of Science and Technology, Hanoi, Vietnam, Hanoi, Vietnam; 2 Graduate University of Science and Technology, Vietnam Academy of Science and Technology, Hanoi, Vietnam; 3 School of Biotechnology and Food Technology, Hanoi University of Science and Technology, Hanoi, Vietnam; 4 Vinh University, Vinh, Vietnam; 5 Vietnam–Russia Tropical Center, Hanoi, Vietnam

**Keywords:** Carbohydrate-active enzymes, *Cellulosimicrobium cellulans*, Levan, Lignocellulose, Termite guts, Whole-genome sequencing

## Abstract

**Background:**

Lignocellulose is a renewable and enormous biomass resource, which can be degraded efficiently by a range of cocktails of carbohydrate-active enzymes secreted by termite gut symbiotic bacteria. There is an urgent need to find enzymes with novel characteristics for improving the conversion processes in the production of lignocellulosic-based products. Although various studies dedicated to the genus *Cellulosimicrobium* as gut symbiont*,* genetic potential related to plant biomass-acting enzymes and exopolysaccharides production has been fully untapped to date.

**Methods:**

The cellulolytic bacterial strain MP1 was isolated from termite guts and identified to the species level by phenotypic, phylogenetic, and genomic analysis. To further explore genes related to cellulose and hemicellulose degradation, the draft genome of strain MP1 was obtained by using whole-genome sequencing, assembly, and annotation through the Illumina platform. Lignocellulose degrading enzymes and levan production in the liquid medium were also examined to shed light on bacterial activities.

**Results:**

Among 65 isolates obtained, the strain MP1 was the most efficient cellulase producer with cellulase activity of 0.65 ± 0.02 IU/ml. The whole genome analysis depicted that strain MP1 consists of a circular chromosome that contained 4,580,223 bp with an average GC content of 73.9%. The genome comprises 23 contigs including 67 rRNA genes, three tRNA genes, a single tmRNA gene, and 4,046 protein-coding sequences. In support of the phenotypic identification, the 16S rRNA gene sequence, average nucleotide identity, and whole-genome-based taxonomic analysis demonstrated that the strain MP1 belongs to the species *Cellulosimicrobium cellulans*. A total of 30 genes related to the degradation of cellulases and hemicellulases were identified in the *C. cellulans* MP1 genome. Of note, the presence of *sacC1-levB-sacC2-ls* operon responsible for levan and levan-type fructooligosaccharides biosynthesis was detected in strain MP1 genome, but not with closely related *C. cellulans* strains, proving this strain to be a potential candidate for further studies. Endoglucanases, exoglucanases, and xylanase were achieved by using cheaply available agro-residues such as rice bran and sugar cane bagasse. The maximum levan production by *C. cellulans* MP1 was 14.8 ± 1.2 g/l after 20 h of cultivation in media containing 200 g/l sucrose. To the best of our knowledge, the present study is the first genome-based analysis of a *Cellulosimicrobium* species which focuses on lignocellulosic enzymes and levan biosynthesis, illustrating that the *C. cellulans* MP1 has a great potential to be an efficient platform for basic research and industrial exploitation.

## Introduction

Termites are social insects contributing to nutrient recycling in terrestrial ecosystems and many vertebrate food chains (Wong et al., 2014). Termites utilize microbes in the hindgut to hydrolyze efficiently cellulose of wood and lignocellulosic materials, into more easily digested sugars and short-chain fatty acids (Ohkuma, 2003; Pasari et al., 2019). As demonstrated previously, termites were not able to digest the lignocellulosic biomass, a mixture of cellulose, hemicellulose, lignin, and pectin completely in the absence of symbiotic microorganisms such as bacteria, archaea (Tokuda & Watanabe, 2007; Wong et al., 2014). Thus, termite gut served as a promising source of identifying novel cellulolytic enzymes as well as an excellent model for further investigating the symbiotic relationships between bacteria and their host.

Recently, bacterial symbionts are acquiring much attention from researchers over the world as potential sources for screening novel and highly efficient lignocellulose-degrading enzymes. They can be applied in many industries like biofuel, food, pulp and paper, and agriculture (Chutani & Sharma, 2016; Pandey, Edgard & Negi, 2016). Species belonging to genus *Bacillus*, *Brevibacillus*, *Cellulomonas*, *Streptomyces*, and *Paenibacillus* were well-studied as excellent cellulases, hemicellulases, xylanase and pectinases producers (Kamsani et al., 2016; Ohkuma, 2003; Pasari et al., 2019; Ventorino et al., 2016). Cellulose hydrolysis is attributed to the synergistic activity of three different groups such as endoglucanase, exoglucanase, and β-glucosidase (Jäger & Büchs, 2012; Wi et al., 2015). Typical hemicellulases are arabinoxylanases, mannanase, and xylanases involved in hemicellulose decomposition (Dashtban, Schraft & Qin, 2009). The whole-genome sequencing approach was used to promote rapid advances in the discovery of potent cellulase and hemicellulose enzymes. In regard to 20 identified CAZymes including 12 endoglucanases, two exoglucanases, and 6 β-glucosidases, halophilic bacterium *Parvularcula flava* NH6-79^T^ serves as cellulolytic enzymes producer (Abdul Karim et al., 2020). *Micromonospora* sp. CP22 was reported to depolymerize the lignocellulosic biomass based on 63 cellulolytic and hemi-cellulolytic CAZymes found in the genome (Chen et al., 2020). The genome *B. velezensis* LC1 was shown to contain 31 genes involved in lignocellulose degradation, and some of these genes were highly induced in presence of bamboo shoot power (Li et al., 2020). This finding is especially significant given that few genomes of termite gut symbiotic bacteria are available.

Levan has received increasing scientific attention due to its application in the pharmaceutical, industrial, and food fields (Belghith et al., 2012; Dahech et al., 2011; Yoo et al., 2004). Indeed, levan is mainly composed of β-2,6 polyfructan with extensive branching through β-(2,1) linkages, that are mostly synthesized by bacterial enzymes (Gojgic-Cvijovic et al., 2019; Mardo et al., 2017). Japan, the US, and South Korea allowed food and pharmaceutical companies to manufacture levan as a functional food additive (Verspreet et al., 2015), while it is not commercially permitted in Europe (Mardo et al., 2017). Bacterial levan is synthesized in sucrose-rich environments through the action of levansucrase (EC 2.4.1.10) for energy reserve and biofilm formation (Shih et al., 2005). Under starvation conditions, accumulated levan was found to be converted into levan-type fructooligosaccharides (L-FOs) that are imported across the outer membrane (Gray et al., 2021). Until now, many bacteria were reported to produce levan such as *Bacillus, Erwinia, Pseudomonas, Microbacterium*, and *Zymomonas* (Gojgic-Cvijovic et al., 2019). The whole genome sequencing shed light on the full potential of levansucrase and levanse in levan-producing bacteria. As revealed in *B. subtilis, sacB* gene encoding for levansucrase catalyzes the synthesis of levan, which is then degraded mainly into levanbiose by the action of levanase such as YveA and YveB. In addition, the *sacB–yveB–yveA* levansucrase tricistronic operon is conserved across 12 complete genome sequences of *B. subtilis* (Dogsa et al., 2013). Interestingly, *sacB* gene was also found to be conserved in halophilic bacterium *Halomonas smyrnensis* AAD6R (Diken et al., 2015).

In previous studies, complete genome sequencing showed that genus *Cellulosimicrobium* is a rich source of glycosidases involved in plant-growth promoting and ginseng biotransformation abilities (Eida et al., 2020; Zheng et al., 2017). To the best of our knowledge, genomic analysis of *Cellulosimicrobium* has not been revealed to prove a better understanding of its genetic basis for other applications. In this study, we report for the first time, the identification and detailed genomic analysis of cellulose-degrading and levan-producing *Cellulosimicrobium cellulans* MP1 isolated from the termite gut. These findings provided a scientific basis for the further employment of strain MP1 and its potential genes in biotechnological processes.

## Materials & Methods

### Isolation of symbiotic cellulolytic bacteria

Drywood termites (*Cryptotermes domesticus*) were collected from rotten tree trunks and bagasse in Nghe An Province, Vietnam. Termites were surface-sterilized using 70% ethanol to remove contamination and then washed with sterile distilled water. The head of each termite was removed using forceps; the entire guts were removed, crushed with glass rods, and subsequently inoculated into 1 ml broth mineral medium M1 (NaNO_3_ 2.5 g; NaCl 0.1 g; KH_2_PO_4_ 2 g; MgSO_4_ 0.2 g; CaCl_2_.6H_2_O 0.1 g, pH 7.0 in a liter) containing 1% carboxymethylcellulose (CMC) or filter paper as a sole carbon source (Gupta, Samant & Sahu, 2012). These cultures were then incubated for 14 days in an incubating sharker at 30 °C. To isolate the cellulolytic bacteria, the growing cultures were spread on the M1 plate medium (K_2_HPO_4_ 1 g; NaNO_3_ 2.5 g; KCl 2 g; peptone 2 g; MgSO_4_ 0.5 g; CMC 10 g; agar 15 g; pH 7.0 in a liter). All bacterial isolates were subsequently purified by re-streaking on the M2 agar plate. Confirmation of the cellulolytic ability of pure isolates was performed on the solid medium by covering the Petri dishes with Congo-red dye (Teather & Wood, 1982). The colonies showing yellow-colored halo zones by Congo-red staining were considered as positive cellulolytic bacteria and the clear zones were measured.

### Physiological, biochemical, and 16S rRNA sequencing analysis

The shape and size of strain MP1 were determined by scanning electron microscope (SEM) JSM-5410 (JEOL, Tokyo, Japan). Gram staining was performed using the conventional methodology and confirmed using the KOH test (Powers, 1995). The effects of different conditions (ranges of pH, NaCl concentration and temperature, carbon and nitrogen sources) on the growth were investigated as previously described (Kamlage, 1996).

Genomic DNA for 16S rRNA gene sequencing was prepared by phenol-chloroform extraction. The amplification of the 16S rRNA gene sequence of strain MP1 was performed by using the universal primer pair 27F (5′ -TAACACATGCAAGTCGAACG-3′) and 1429R (5′-GGTGTGACGGGCGGTGTGTA-3′) (Phi et al., 2010). A sequence similarity search was carried out using the BLAST program (http://blast.ncbi.nlm.nih.gov/Blast.cgi). The phylogenetic tree was computed by using the neighbor-joining method with 1,000 bootstrap in MEGA version 6.0 (Tamura et al., 2013). Numbers at nodes indicate percentages of 1000 bootstrap re-samplings and *Bifidobacterium bifidum* DSM 20456 (S83624) was used as the outgroup branch. The 16S rDNA gene sequence of strain MP1 was deposited onto the GenBank (NCBI) under accession number MW534740.

### Genome sequencing, *de novo* assembly, and annotation

For library construction, DNA was extracted from a pure culture of a single colony of strain MP1 using G-spin™ Total DNA Extraction Mini Kit according to the manufacturer’s instructions. The quantity and quality of extracted DNA were evaluated by electrophoresis in 0.6% (w/v) agarose gel and NanoDrop spectrophotometer 2000 Thermo Scientific in order to construct a paired-end library. The constructed genome library was then sequenced using the Illumina platform (Illumina, California, USA). Quality control and read trimming were conducted using FastQC version 0.11.5 (http://www.bioinformatics.babraham.ac.uk/projects/fastqc) and Trimmomatic version 0.36 (Bolger, Lohse & Usadel, 2014). The *de novo* genome assembly was made with SPAdes v.3.13 (Bankevich et al., 2012), which was then analyzed for its completeness using the Benchmarking Universal Single-Copy Orthologous (BUSCO) version 3 (https://gitlab.com/ezlab/busco).

The draft genome assembled into contigs was annotated using Prokaryotic Genomes Annotation Pipeline (PGAP; http://www.ncbi.nlm.nih.gov/genome/annotation_prok/) at NCBI. In addition, the CRISPRCasFinder was used to identify putative CRISPR loci and Cas cluster (Grissa, Vergnaud & Pourcel, 2007). Orthologous genes and Gene ontology (GO) were analyzed using clusters of orthologous genes (COGs) (Galperin et al., 2015) and InterProScan 5 (Jones et al., 2014), respectively. Virulence factors-encoding genes were identified using the Pathosystems Resource Integration Center (PATRIC) platform (Wattam et al., 2017). The graphical map of the circular genome was also generated using PATRIC. The draft genome sequence was deposited in the GenBank (NCBI) database under accession number: JAFGYF000000000.

### Analysis of whole-genome similarity

To classify strain MP1 at the species level, whole-genome similarity including average nucleotide identity (ANI) calculation and digital DNA–DNA hybridization (dDDH) was performed. The ANI was calculated using the orthologous average nucleotide identity (OrthoANI) (Lee et al., 2016). The MP1 genome sequence data was uploaded to the Type (Strain) Genome Server (TYGS) for a whole-genome-based taxonomic analysis (https://tygs.dsmz.de). *In silico* dDDH and the branch lengths were and the Genome BLAST Distance Phylogeneny evaluated using Genome-to-Genome Distance Calculator (GGDC) (Meier-Kolthoff et al., 2013).

### Comparative genomics and prediction of Carbohydrate-active enzyme

A genome-wide comparison of COGs between the assembled genome of *Cellulosimicrobium cellulans* MP1 and three other *C. cellulans* available in GenBank, including J36 (NZ_JAGJ01000000.1), LMG16121 (NZ_CAOI01000000.1), and ZKA 48 (NZ_QUMZ01000000.1) was implemented using the OrthoVenn web server with default parameters (*E*-value 1e−5 and inflation value 1.5) (Wang et al., 2015). The putative genes encoding CAZymes in the comparative genomes were predicted using the dbCAN2 meta server and classified by DIAMOND, HMMER, and Hotpep via CAZy and dbCAN databases, respectively. The top hits with e-value <1E−17, minimum homology rate >50%, and coverage >45% were considered to be homologs.

### Determination of extracellular enzymatic activities

To verify the production of endoglucanase, *C. cellulans* MP1 was cultivated in the TN medium (rice bran 20 g; soya peptone 10 g, casein 10 g, NaCl 1 g, pH 7.0 in a liter) at 37 °C with vigorous sharking. At different *time intervals*, samples were taken, followed by centrifugation at 4 °C, 12,000 rpm for 10 min to remove bacterial cells and insoluble materials from the culture broth. About 0.5 ml of the crude enzyme solution was added into 0.5 ml of 0.05 M sodium phosphate, pH 7.0 buffer containing 1% of CMC. The mixture was then incubated at 30 °C for 30 min, which was stopped by adding 1 ml of 3,5′–dinitrosalicylic acid (DNS) reagent followed by boiling the reaction mixture at 100 °C for 5 min (Miller, 1959). As for xylanase production, strain MP1 was cultivated on M1 medium (K_2_HPO_4_ 1 g; KCl 2 g; NH_4_Cl 2.5 g; yeast extract 2 g, MgSO_4_ 0.5 g, sugar cane bagasse 5 g, pH 7.0 in a liter) at 37 °C for 4 days. At different *time intervals*, culture was centrifuged at 4 °C, 12,000 rpm for 10 min to obtain the crude enzyme. About 0.5 ml of the crude enzyme was mixed with 0.5 ml of the substrate solution and incubated at 50 °C for 5 min, followed by adding 1 ml DNS reagent. Xylanase activity was assayed using 1% birchwood xylan solution in acetate buffer, pH 5.0 as the substrate (Bailey, Biely & Poutanen, 1992). To quantify pectinase activity, the M2 medium (NH_4_Cl 2.5 g; K_2_HPO_4_ 1 g; MgSO_4_ 0.5 g; KCl 2 g; yeast extract 2 g, rice bran 10 g; lactose 10 g; pH 7.0 in a liter) was used. The pectinase assay was performed at 40 °C for 30 min as described previously (Mercimek Takcı& Turkmen, 2016). The quantitative enzyme assays of endoglucanase, xylanase, and pectinase were performed according to standard IUPAC procedures and expressed as international units (IU) (Ghose, 1987). One unit (IU) of enzymatic activity is defined as the amount of enzyme that releases 1 µmol reducing sugars per ml of culture supernatant per minute under assay conditions.

### Levan determination

To test the ability to synthesize levan, the *C. cellulans* MP1 was cultured overnight in batch culture medium (yeast extract 7 g, (NH_4_)_2_SO_4_ 1.5 g, K_2_HPO_4_ 2.5 g, pH 7.0 *in a liter*) at 37 °C under vigorous agitation. The overnight culture was transferred to a new batch culture medium supplemented with 100 and 200 g/l sucrose and adjusted to an optical density at 600 nm of 0.1. The culture was centrifuged at 10,000 rpm for 10 min at different time intervals. Cell-free supernatant was used to determine levan produced during fermentation (Gojgic-Cvijovic et al., 2019). Levan was harvested by adding three volumes of ice-cold ethanol. The mixture was kept at 4 °C for 12 h, centrifuged at 12,000 rpm at 4 °C for 20 min, and washed with 75% ethanol to remove the residual sugars. The obtained precipitate was hydrolyzed with 0.1 M HCl at 100 °C for 1 h. After neutralizing with 2 M NaOH, levan content was determined according to Somogyi and Nelson (Somogyi, 1945).

## Results

### Cellulose-degrading potential and identification of the strain MP1

Of the 65 isolates able to grow on CMC plates as the sole carbon source, a total of 8 bacterial isolates produced variable zones of CMC clearance after Congo-red staining. Among those, MP1 was selected due to showing the maximum zone of clearance (9 ± 1, 1 mm). In support of this result, enzyme assays for cellulase activity on CMC and filter paper were found to be the highest for MP1 with 0.66 ± 0.15 IU/ml after 48 h and 0.33 ± 0.05 FPU/ml after 72 h of cultivation, respectively (Table S1). These results indicated that isolate MP1 is a potent cellulolytic bacterium for further study.

**Figure 1:**
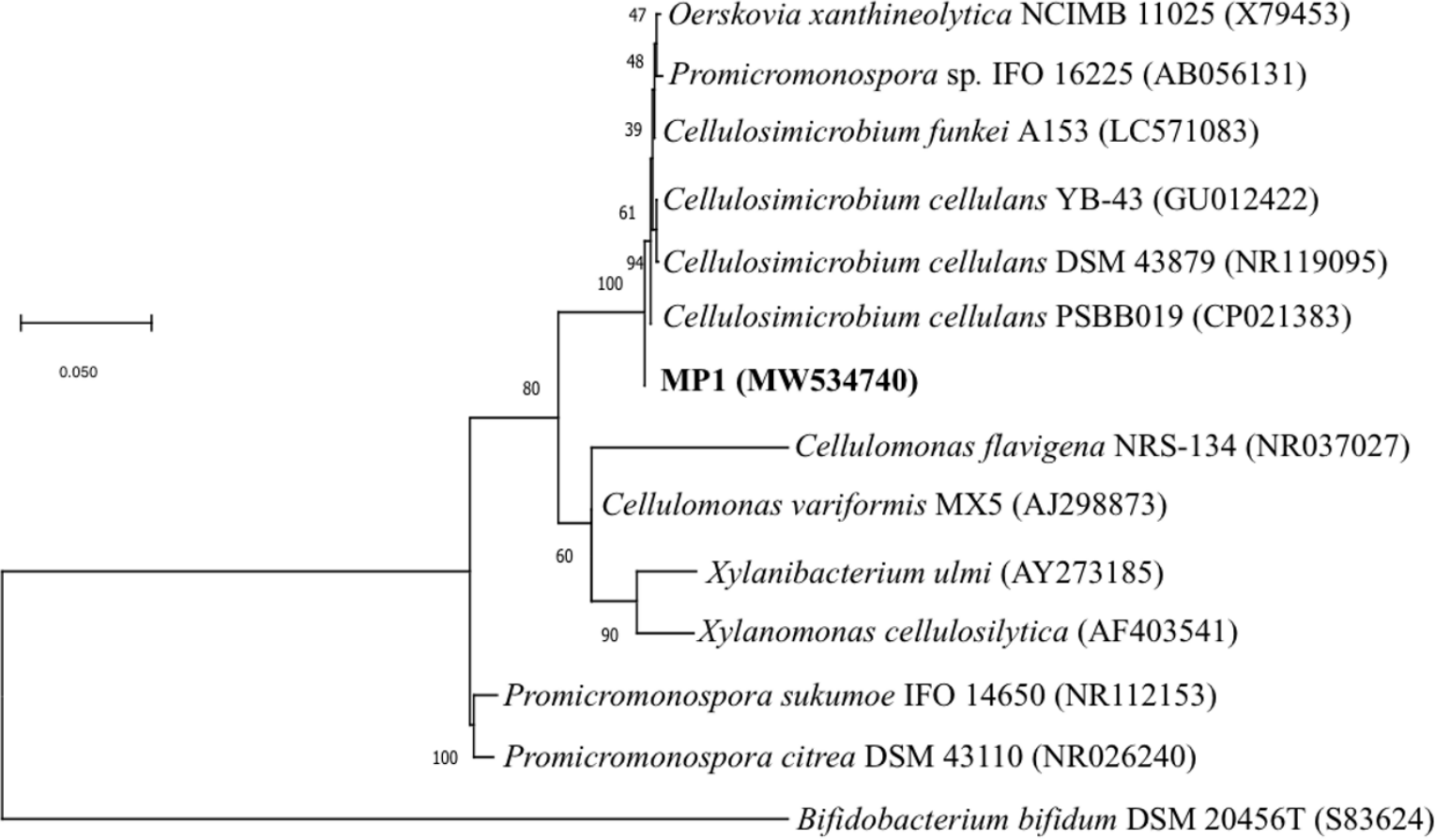
Identification of the strain MP1. The Maximum-likelihood phylogenetic tree based the 16S rRNA sequence of strain MP1 and representatives of reference type strains. Bar: 0.05 substitutions per site.

**Figure 2:**
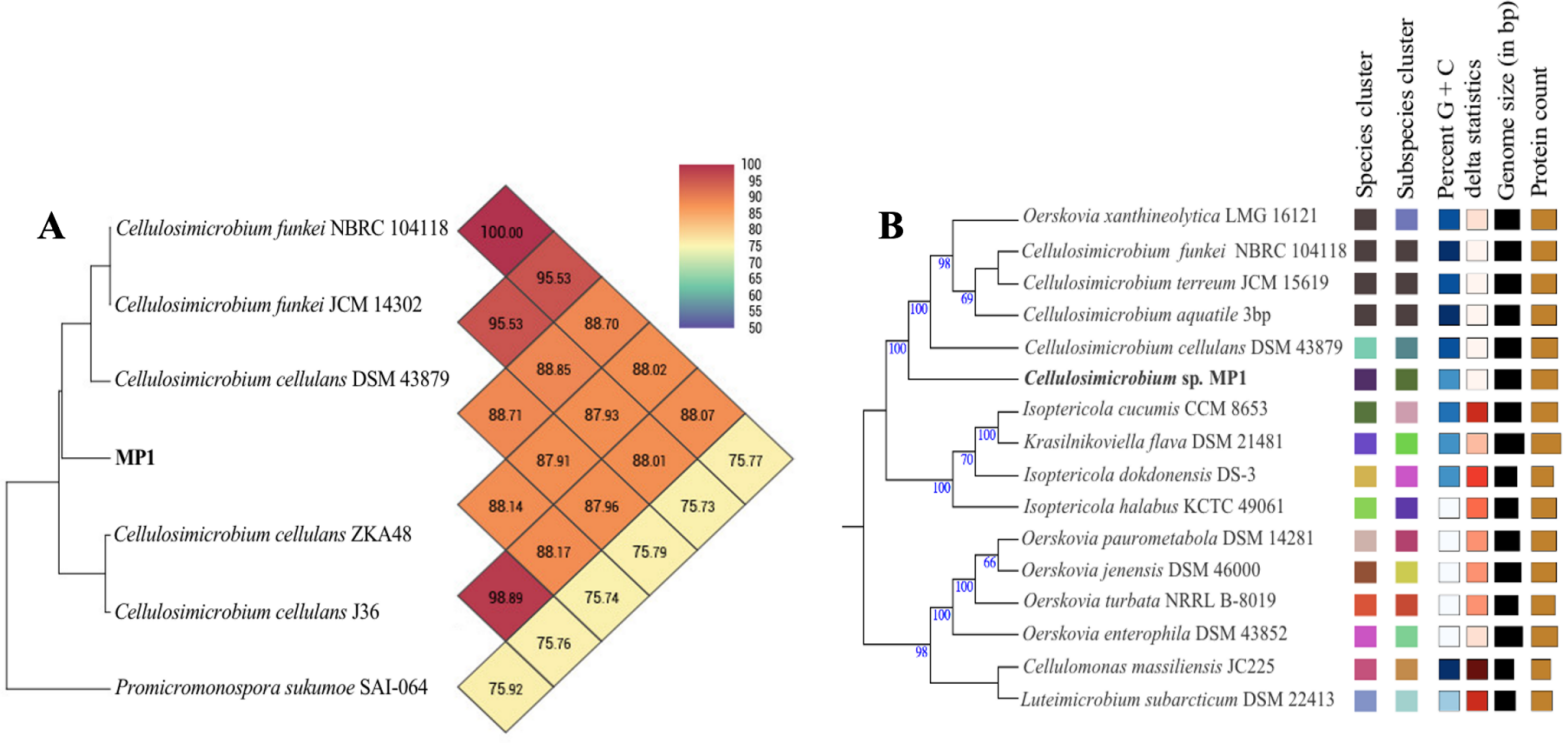
Phylogenomic classification of *Cellulosimicrobium* sp. MP1 based on genome analysis. (A) Heatmap of OrthoANI values for *Cellulosimicrobium* sp. MP1 and five closely related species. (B) Genome Basic Local Alignment Search Tool (BLAST) distance phylogenies (GBDP) using Type Strain Genome Server (TYGS) platform. Branch lengths are scaled in terms of GBDP distance formula *d*5. Numbers above branches are GBDP pseudo-bootstrap support values > 60% from 100 replications, with an average branch support of 84.3%. Tree was rooted at midpoint.

The phenotypic examinations indicated that MP1 grew well on LB agar after 2 days of incubation at 37 °C, producing colonies that were circular, smooth, convex, and pale yellow in colour. MP1 cells were Gram-positive, non-spore-forming rods, and nonmotile. The strain MP1 could utilize D-galactose, D-sucrose, D-raffinose, and amygdalin as sole carbon and energy (Table S2). It gave a positive test for catalase, starch hydrolysis, β-galactosidase, and nitrate reduction, whereas negative for oxidase, gelatinase, urease, indole, H_2_S, acetoin. The 16S rDNA gene sequence of MP1 was aligned with the similar nucleotide sequences in the GenBank database in which a phylogenetic tree was then constructed. The neighbor-joining phylogenetic tree showed the close relationship between MP1 and related *Cellulosimicrobium* species and the highest homology to *Cellulosimicrobium cellulans* DSM 43879 (99.5%) and *Cellulosimicrobium funkei* A153 (99.6%) (Fig. 1)*.* Further, the OrthoANI software was performed to determine the OrthoANI value between isolate MP1 and five closely related *Cellulosimicrobium* species. It revealed that MP1 shared high similarity to *C. cellulans* DSM 43879 (88.71%), *C. funkei* JCM 14302 (91.35%), and *C. funkei* JCM NRBC 104118 (91.35%), and low nucleotide similarity was observed with *Promicromonospora sukumoe* SAI-064 that were out of distinct sub-clade (Fig. 2A). This result confirmed that this strain was not considered to be a novel species.

**Figure 3:**
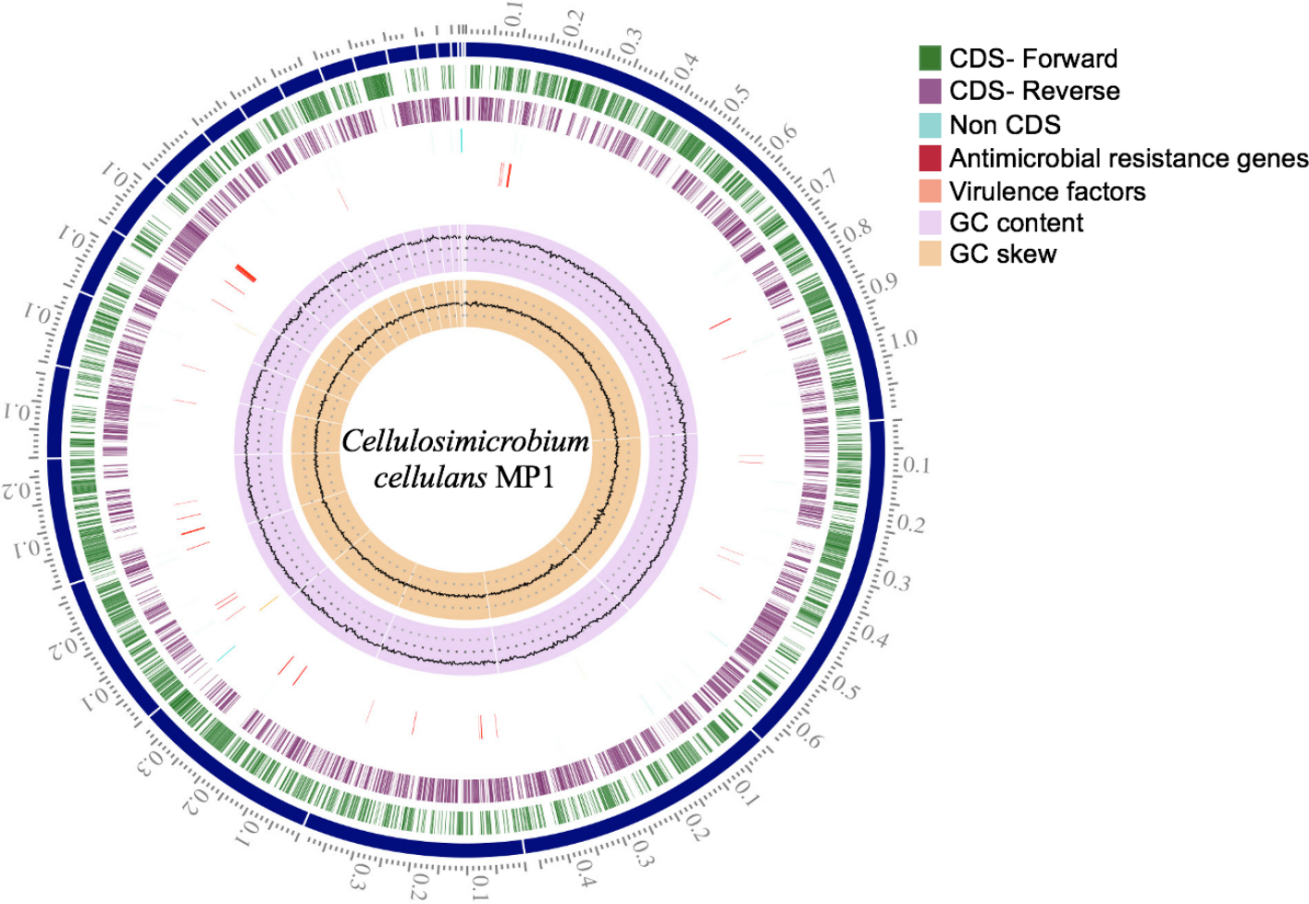
Circular genome map of *C. cellulans* MP1.

To make identification more accurate at the species level, the whole-genome-based taxonomic analysis conducted by the Type Strain Genome Server (TYGS) platform suggested that *Cellulosimicrobium* sp. MP1 was closest to *C. cellulans* DSM 43879 with digital DNA-DNA hybridization (dDDH) values and differences in guanine-cytosine (GC) content of 57.5% (formula d_6_) and 0.56%, respectively (Fig. 2B). As shown previously, the differences between *C. funkei* and *C. cellulans* in phenotypic characteristics are motility and ability to utilize raffinose (Hamada et al., 2016; Yoon et al., 2007). Based on the phenotypic characteristics and genome-wide comparison, this strain was identified as *Cellulosimicrobium cellulans* MP1. This bacterium was deposited at VAST-Culture Collection of Microorganisms (VCCM) with the accession number VCCM 14150.

### Genome sequence and general features of the *C. cellulans* strain MP1

Briefly, the standard short insert library yielded 443,986,169 bases resulting in 4,487,842 mapped reads with about 92.2-fold sequencing depth. The draft genome of strain MP1 (4,580,223 bp with GC content of 73.9%) was assembled into a ring chromosome, comprising of 23 contigs and no plasmid was detected (Fig. 3). The genome was predicted to have 4,088 genes assigned for 3,964 protein-coding sequences (CDS), 61 rRNA sequences, 55 tRNA sequences, 3 ncRNA sequences (Table 1). Moreover, a total of three virulence factors present in *C. cellulans* MP1 included dihydroxy-acid dehydratase, CarD-like transcriptional factor, and two calmodulin-like proteins (Fig. 3).

**Figure 4:**
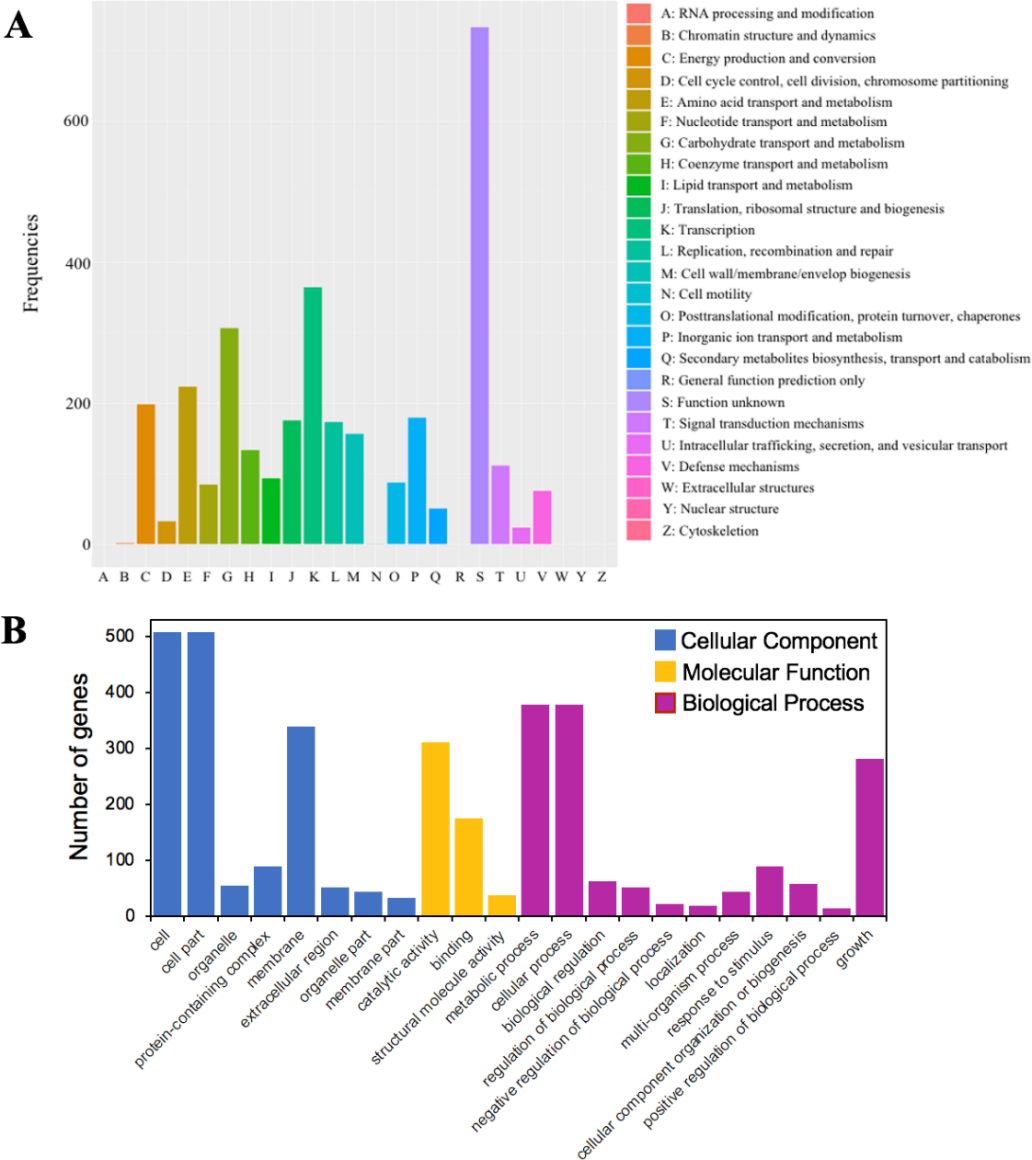
The functional annotations of *C. cellulans* MP1. (A) Cluster of orthologous gene (COG) classification. (B) Gene ontology (GO) functional classification.

A total of 79.5% (3,216 out of 4,046) of the protein-coding sequences were assigned to 21 out of 25 COG functional categories (Fig. 4A). Transcription (K: 365 protein-coding sequences), carbohydrate transport and metabolism (G: 307), amino acid transport and metabolism (E: 224), energy production and conversion (C: 199), and inorganic ion transport and metabolism (P: 180) were found to be the largest categories. By contrast, the least represent groups included cell cycle control, cell division, chromosome partitioning (D: 33), chromatin structure and dynamics (B: 2), and cell motility (N: 1).

**Table 1:** Genomic features of *C. cellulans* MP1.

**Features**	**Chromosome**
Genome size (bp)	4,580,223
No. of contigs	23
No. of plasmids	0
G + C content (%)	73.9
Genes (total)	4,088
CDSs (coding)	3,964
rRNAs	61
tRNAs	55
ncRNAs	3
Pseudogenes	63
CRISPRS	0
GenBank accession number	JAFGYF000000000

GO analysis was used to provide a deeper understanding of the functional catalogs of strain MP1. A total of 3,165 genes were assigned to 43 subclasses, including 13 subclasses of the cellular component (CC) class, 10 subclasses of the molecular function (MF) class, and 20 subclasses of the biological process (BP) class (Fig. 4B). In detail, the CC class occupied the most genes (1638 genes; 45.3%), followed by the BP (1424 genes; 39.4%), and MF (553 genes; 15.3%) class. The most abundant pathways were cell (GO:0005623; 508 genes), cell part (GO:0044464; 508 genes), and membrane (GO:0016020; 340 genes), which could be considered as the main functional groups of genes belonging to the CC class. Within the BP class, the three most prevalent molecular functions were metabolic process (GO:0008152; 378 genes), cellular process (GO:0009987; 379 genes), and growth (GO:0040007; 281 genes) (Fig. 4B). Among all subclasses belonging to the MF class, catalytic activity (GO:0003824) and binding (GO:0005488) contributed the most genes, with 311 and 176, respectively.

### Genome-wide comparison of COGs and Carbohydrate-active enzymes

**Figure 5:**
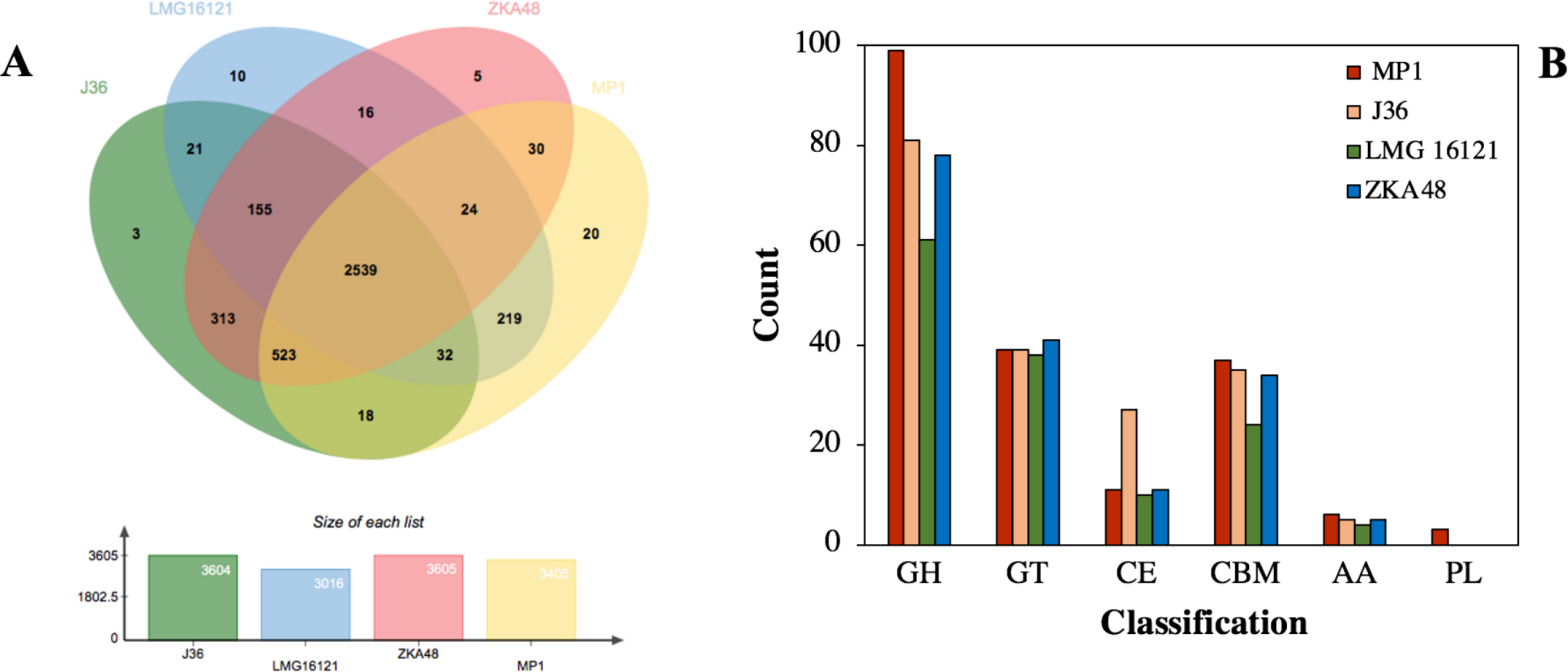
Comparative genomes between *C. cellulans* MP1 and three other *C. cellulans* strains. (A) Venn diagram represents the numbers of unique and shared orthologous genes of each strain. (B) Comparative genomic analysis of CAZymes across *C. cellulans* strains.

Given that microbial evolution is due to vertical descent from a single ancestral gene leading to orthologous genes in different species, it is necessary to explore gene function, gene structure, and molecular evolution by using a genome-wide comparison of COGs in different strains. The COGs of *C. cellulans* MP1 were compared with four other strains, including J36, LMG16121, and ZKA48. It showed that *C. cellulans* MP1 comprised of 3405 COGs and 794 singletons. *C. cellulans* J36 and ZKA48 included 3604 and 3605 COGs, respectively, whereas the lowest COGs were observed in LMG16121 (Fig. 5A). Surprisingly, strain MP1 had the largest number of singletons (*n* = 794). Venn diagram denoted that a total of 2,539 COGs were commonly shared by all four strains of *C. cellulans*. Among the unique COGs observed in all strains, strain MP1 had the largest number of 20 (Fig. 5A). To support this result, the REALPHY phylogeny builder web tool was used to compare *C. cellulans* genomes. A maximum-likelihood phylogenetic tree showed that MP1 and LMG16121 formed a monophyletic clade, suggesting a strong evolutionary relationship (Fig. S1).

Using dbCAN 2 meta server, the putative genes encoded for CAZymes present in *C. cellulans* MP1 were screened to find out the genes responsible for cellulose and hemicellulose degradation. After removing the sequences that did not meet the filtering criteria, a total of 195 predicted CAZymes was identified corresponding to 4.8% of the total of 3,216 protein-coding sequences observed in this strain. Glycoside hydrolases (GHs) involved in the degradation of the most plant biomass such as cellulose and hemicellulose were predicted to be the most abundant subfamily with 99 enzymes. Next, 39 glycosyltransferases (GTs), 37 carbohydrate-binding modules (CBMs), 11 carbohydrate esterases (CEs), 6 enzymes with auxiliary activities (AAs), and 3 polysaccharide lyases (PLs) were detected (Fig. 5B).

The genome of *C. cellulans* MP1 was compared to closely related *C. cellulans* such as *C. cellulans* J36, *C. cellulans* LMG16121, *C. cellulans* ZKA48. Generally, *C. cellulans* MP1 had significantly more CAZyme domains found than other *C. cellulans* strains. Despite forming a monophyletic clade with strain LMG16121, MP1 was 56 CAZyme domains higher than LMG16121. Meanwhile, with the exception of LMG16121, the family numbers of GT, CBM, and AA family numbers were the same in all compared strains, suggesting the coexistence of these genes in breaking down cellulosic biomass (Fig. 5B).

### Mining of plant biomass-acting enzymes in *C. cellulans* MP1 gene pool

CAZyme analysis suggested that 30 cellulose-related sequences were detected in the genome of *C. cellulans* MP1. The major families related to the degradation of cellulose are GH6, GH9, GH48, GH10, GH16, GH1, GH3, GH13, and GH64. Based on the annotation, 5 endoglucanases (two GH6 and three GH9), 3 exoglucanases (GH6, GH10, and GH48), and lichenase (GH16) were revealed in the genome of strain MP1 (Table 2). A total of five out of the eight annotated endoglucanases and exoglucanases (*orf_454, orf_1616, orf_3244, orf_1607, orf_1611*) contained a CBM2 domain appended to them, which have been known to bind to crystalline cellulose, insoluble chitin, and xylan (McLean et al., 2002). CBM2 was the major CBMs present in the genome of strain MP1. In the β-glucosidases family, three GH1 and six GH3 were considered as other important members for cellulose degradation and three GH13 had α-glucosidase activity. It is interesting to note that two pectate lyases such as PL4 (*orf_102*) and PL1 (*orf_1326*) belonging to the large class of PL were identified (Table 2). Given that pectin is involved in providing structural support for plant such as cell walls, wall integrity, and cell–cell cohesion, pectate lyases (EC 4.2.2.2) belonging to pectinase catalyze the eliminative cleavage of α-1,4-glycosidic bonds between C_4_ and C_5_ of pectin or pectic acid, producing unsaturated methyloligogalacturonates (Abbott, Gilbert & Boraston, 2010; Hugouvieux-Cotte-Pattat, Condemine & Shevchik, 2014). Pectate lyases are important symbiotic bacteria through facilitating their growth in presence of highly pectinolytic bacteria and under pectin-rich environments (Hugouvieux-Cotte-Pattat, Condemine & Shevchik, 2014).

**Table 2:** List of predicted cellulolytic and hemicellulolytic enzymes present in the genome of *C. cellulans* MP1.

**Classification**	**Locus tag**	**Predicted function**
Cellulose-related	Orf_454, Orf_1616, Orf_2130, Orf_2755, Orf_3244	Endoglucanase [EC 3.2.1.4]
	Orf_1607, Orf_1610, Orf_1611	Exoglucanase [EC 3.2.1.91]
	Orf_2289	Lichenase [EC 3.2.1.73]
	Orf_2294, Orf_2388, Orf_2464, Orf_2606, Orf_3385, Orf_401, Orf_403, Orf_404, Orf_922	β-glucosidase [EC 3.2.1.21]
	Orf_2704, Orf_2893	Oligo-1,6-glucosidase [EC 3.2.1.10]
	Orf_2898	Maltodextrin glucosidase [EC 3.2.1.20]
	Orf_802	Glucan endo-1,3- β-glucosidase [EC 3.2.1.39]
	Orf_3703	β-galactosidase [EC 3.2.1.23]
	Orf_2449	Trehalose-6-phosphate hydrolase [EC 3.2.1.93]
	Orf_102	Pectate trisaccharide-lyase [EC 4.2.2.22]
	Orf_1326	Pectate lyase [EC 4.2.2.2]
	Orf_19, Orf_21	Levanase [EC 3.2.1.80]
	Orf_20	Levanbiose-producing levanase [EC 3.2.1.64]
	Orf_22	Levansucrase [EC 2.4.1.10]
Hemicellulose-related	Orf_905	Mannan endo-1,4- β-mannosidase [EC 3.2.1.78]
	Orf_3988, Orf_4004	Bifunctional β-xylosidase/ α-arabinosidase [EC 3.2.1.37; EC 3.2.1.55]
	Orf_4003	Arabinoxylan arabinofuranohydrolase [EC 3.2.1.55]
	Orf_35, Orf_2605	α-xylosidase [EC 3.2.1.177]
	Orf_24, Orf_2083, Orf_3386, Orf_3999, Orf_4000, Orf_4002	α-L-arabinofuranosidase [EC 3.2.1.55]
	Orf_34, Orf_3650, Orf_4001	Non-reducing end β-L-arabinofuranosidase [EC 3.1.1.185]
	Orf_3698	Exo- α-(1->6)-L-arabinofuranosidase [EC 3.2.1.-]
	Orf_1000, Orf_2117, Orf_3772	Endo-1,4- β-xylanase [EC 3.2.1.8]
	Orf_266, Orf_2247	α-galactosidase [EC 3.2.1.22]

For hemicellulose degrading genes mining, a total of 21 annotated proteins were deduced to involve in hemicellulose degradation. The major families responsible for the breakdown of hemicellulose were GH43, GH31, GH127, GH51, GH10, GH36, and GH4, and the total number of enzymes, including all families, was 21 (Table 2). Among them, the most abundant enzymes were attributed to GH43 (9 enzymes), followed by GH124 (3 enzymes) and GH31 (2 enzymes), indicating that strain MP1 might have great potentials for degradation of hemicellulosic backbones or debranding hemicellulose. Hemicellulose-related genes were predicted as mannan endo-1,4-β-mannosidase, arabinoxylan arabinofuranohydrolase, α-xylosidase, α-L-arabinofuranosidase, β-L-arabinofuranosidase, endo-1,4-β-xylanase, α-galactosidase, and bifunctional β-xylosidase/ α-arabinosidase (Table 2). Only two enzymes, mannan endo-1,4-β-mannosidase and α-galactosidase, have the ability to hydrolyse parts of mannan, the second major component in hemicellulose. Two endo-1,4-β-xylanase (*orf_100* and *orf_3772*) and one bifunctional β-xylosidase/ α-arabinosidase (*orf_3988*) were coupled with either CBM2 or CBM9. Surprisingly, genes encoding arabinofuranosidase (*orf_3999*, *orf_4000*, *orf_4001* and *orf_4002*), arabinoxylan arabinofuranohydrolase (*orf_4003*), and bifunctional β-xylosidase/ α-arabinosidase (*orf_4004*) are clustered in an operon. However, AA enzymes were not found to be related to cellulose and hemicellulose degradation.

**Figure 6:**
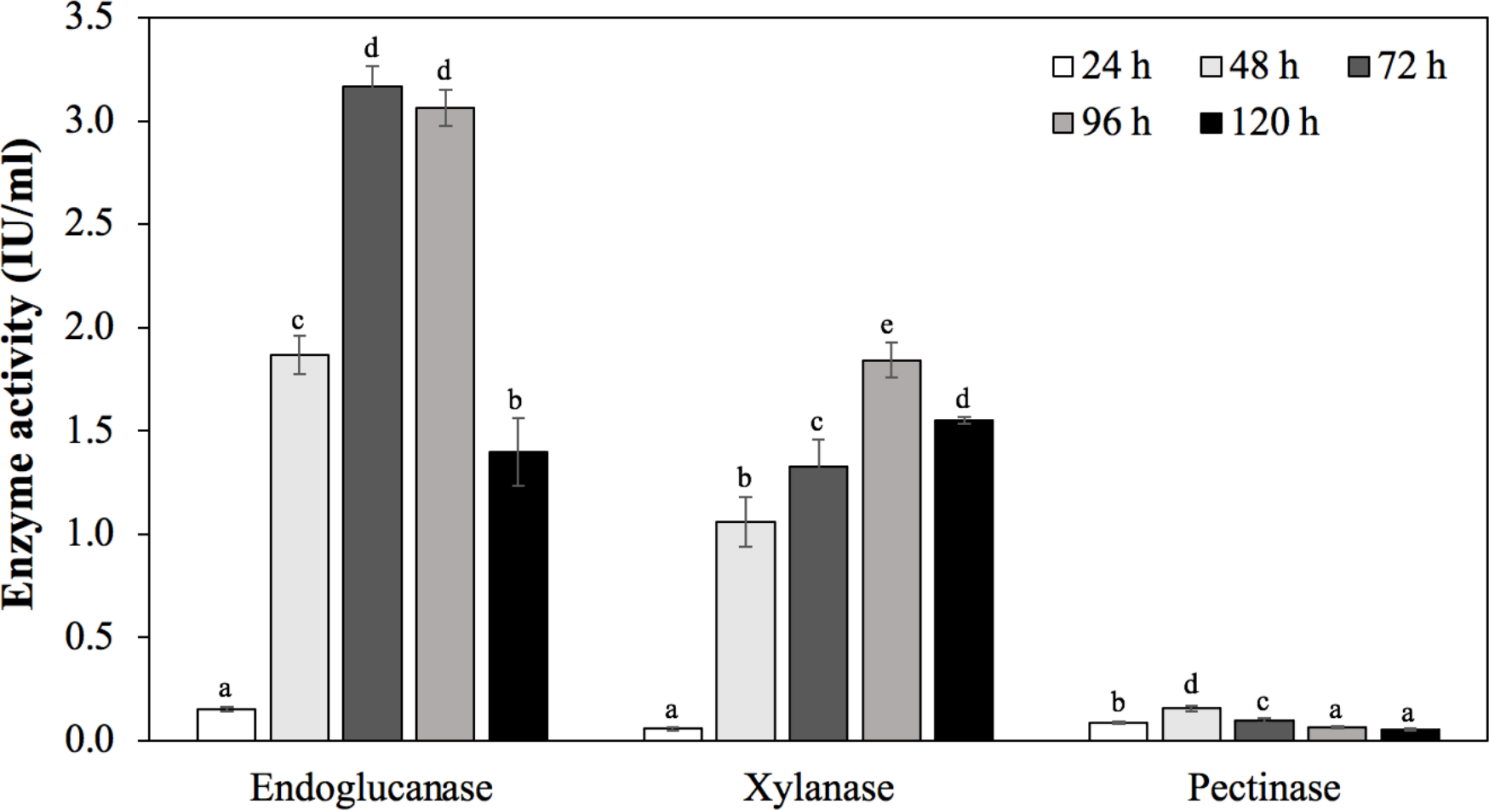
Enzymatic activities of *C. cellulans* MP1 observed in different incubation periods. Mean values with different letters a-d are significantly different according to the Fisher LCD test (*P* < 0.05).

To confirm the ability to produce endoglucanase, xylanase, and pectinase of strain MP1, the quantitative enzyme assay was monitored across different incubation periods (24 h, 48 h, 72 h, 96 h, 120 h) (Fig. 6). Using rice bran as an inducible substrate for endoglucanase production, the lowest endoglucanase activity of 0.15 ± 0.01 IU/ml was observed at 24 h, followed by an increase of activity within 48 h to 96 h. The highest endoglucanase production of about 3.17 ± 0.10 IU/ml was achieved after 72 h of incubation. The reduced activity of endoglucanase was notified as 1.40 ± 0.17 IU/ml at 120 h. Xylanase activity of strain MP1 was monitored on M1 medium supplemented with sugar cane bagasse at 37 ° C for 120 h. The result was a steep increase within 0 to 72 h of incubation times. The highest enzyme production was recorded during the stationary phase reaching maximum (1.84 ± 0.08 IU/ml) at 96 h, followed by a slight decrease after 120 h (Fig. 6C). Regarding to pectinase, the maximum pectinase yield was 0.16 ± 0.01 IU/ml after 48 h of incubation. Increasing incubation time was subjected to a significant decrease of pectinase activity. Despite the fact that a number of studies involved in the yields of endoglucanase, xylanase, and pectinase have been performed, it is hard to compare due to the effect of production conditions, substrate and assay conditions, and the way of defining the units.

### Levan exopolysaccharide biosynthesis and degradation

**Figure 7:**
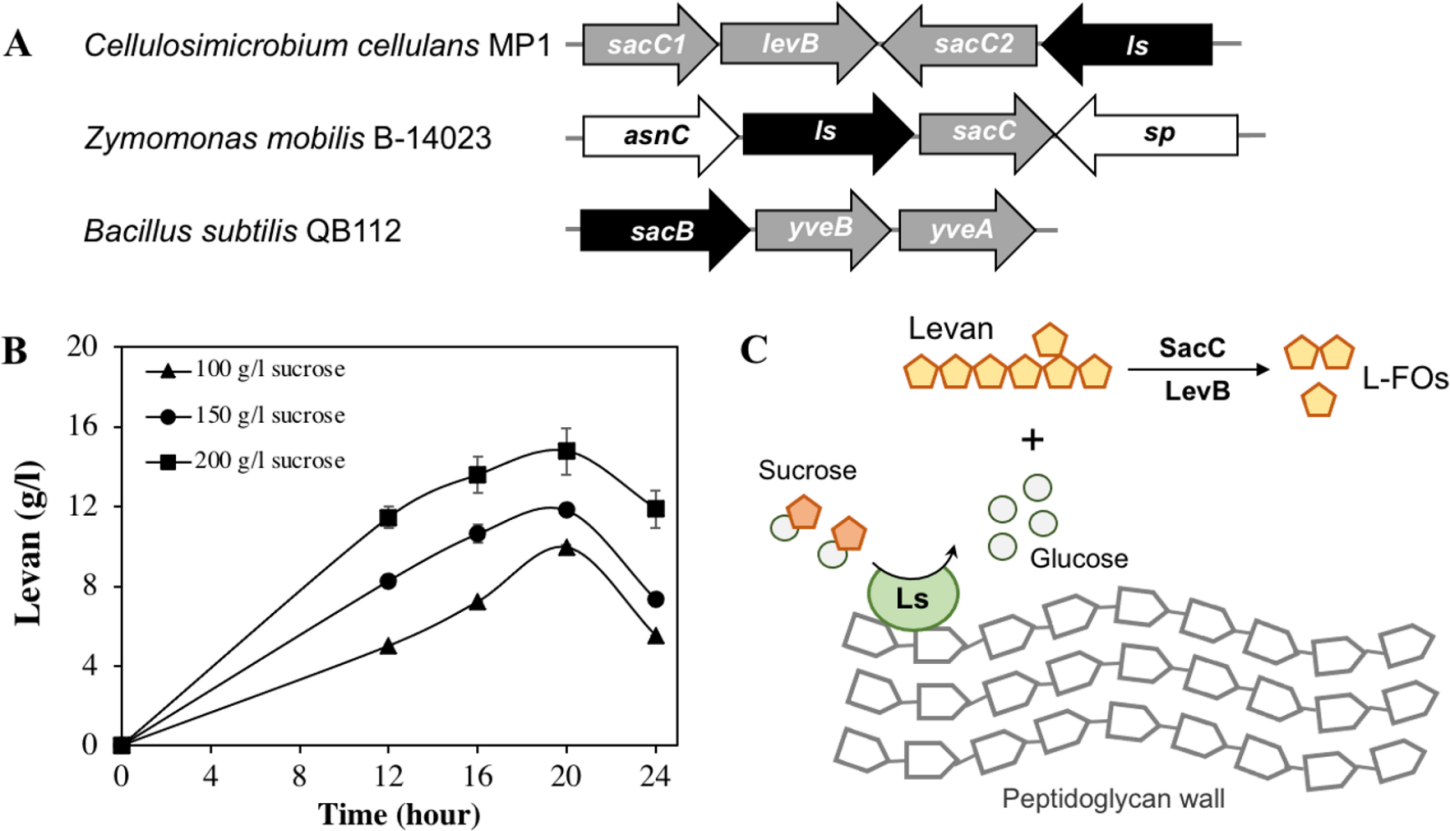
Levan biosynthesis in *C. cellulans* MP1. (A) Genetic organization of levan operon in *C. cellulans* MP1, *Zymomonas mobilis* B-14023** and *Bacillus subtilis* QB112. The *ls* and *sacC-levB* homologous genes are denoted in black and gray, respectively. (B) Time course of levan production. (C) Proposed mechanism for levan and levan-type fructooligosaccharide (L-FOs) biosynthesis.

Apart from cellulose-related genes, we were interested in the gene encoding for levansucrase (*orf_22*) since the ability of the genus *Cellulosimicrobium* to produce levan has not been studied yet. Levansucrase (EC 2.4.1.10) belonging to GH32 participates in synthesizing levan using high sucrose concentration as the main substrate (Raga-Carbajal et al., 2018). The *C. cellulans* MP1 *ls* gene consists of 1,851 nucleotides encoding a protein of 616 amino acids with the predicted molecular mass of 66.6 kDa. Alignment of the amino acid sequence of *C. cellulans* MP1 Ls shows the highest level of identity with *Gluconacetobacter diazotrophicus* (61%) and *Microbacterium saccharophilum* (58%) (Fig. S2). In addition, levansucrase is clustered alongside levanbiose-producing levanase (*levB*-*orf_20*) and levanases (sac*C 1*-*orf_19* and *sacC 2*-*orf_21*) in an operon (Fig. 7A). This result suggested that levan produced by *C. cellulans* MP1 may be hydrolyzed into L-FOs such as levanbiose and levanases. It is noteworthy that the *sacC1-levB-sacC2-ls* operon was not present in the compared *C. cellulans* strains

The levan production in media with different initial sucrose concentrations was monitored as shown in Fig. 7B. Levan production depended on cultivation time and sucrose concentration. In the medium with 100 g/l sucrose, the maximum levan concentration was achieved at 20 h (9.9 ± 0.11 g/l), followed by a significant decrease after 24 h (5.5 ± 0.02 g/l). When sucrose concentration was increased to 200 g/l, the highest yield of levan was 14.8 ± 1.2 at 20 h. It was noteworthy that levan was accumulated in the stationary phase.

## Discussion

More recently, the exploitation of new plant biomass-acting enzymes and exopolysaccharides with special characteristics has become important since the technological use of agro-industrial residues is increasing. They are exploited favourably in food, paper, cosmetic and pharmaceutical industries (Angelin & Kavitha, 2020; Walia et al., 2017; Zheng et al., 2017). In this context, termites are thought to rely on the gut microbiome to digest wood and other types of plant biomass consisting mainly of cellulose as well as hemicellulose (Calusinska et al., 2020), making them an ideal source to search for new enzymes. Many cellulolytic bacteria from termite have been extensively investigated in the past, despite difficulties in isolation and cultivation (Kamsani et al., 2016). However, despite the use of high-throughput next-generation sequencing, potent biomass-acting enzymes have yet to be revealed. Our work is the first genome analysis systematically elucidating enzymes related to the decomposition of cellulose and hemicellulose and levan production of termite-associated *C. cellulans*. The findings provide valuable genome information for biotechnological applications.

Despite having potential in many fields, the genus *Cellulosimicrobium* remains poorly investigated. To date, the genus *Cellulosimicrobium* consists 7 species (six validly and one non-validly published names): *C. variabile* (Bakalidou et al., 2002), *C. funkei* (Brown et al., 2006), *C. cellulans* (Schumann, Weiss & Stackebrandt, 2001), *C. terreum* (Yoon et al., 2007), *C. marium* (Hamada et al., 2016), *C. aquatile* (Sultanpuram et al., 2015), and *C. arenosum* (Oh et al., 2018). Only *C. variabile* was reportedly isolated from the hindgut of termites (Qin et al., 2018; Sharma, Gilbert & Lal, 2016). Since *C. cellulans* MP1 produced highly active cellulolytic activity on CMC and filter paper, we subsequently analyzed its whole genome for the presence of lignocellulose-degrading CAZymes. Of note, the genome possesses 17 cellulolytic and 21 hemicellulolytic enzymes, in which a total of 11 enzymes featuring CBM domains (CBM2, CBM4, CBM6, CBM9, CBM35) were found. CBM domains play an important role in enhancing the substrate-binding capability of the enzymes, and some CBMs are able to enhance the thermostability of the enzyme (Gilbert, Knox & Boraston, 2013). CBM2 binds to various GH families including GH6 (*orf_454, orf_3244*), GH9 (*orf_1616*), GH10 (*orf_1000, orf_1611*), GH43 (*orf_3988*), and GH48 (*orf_1607*), which indicates the ability of these enzymes to bind to and support catalytic domains to hydrolyze crystalline cellulose and xylan. CBM9 domain was found only in endo-1,4- β-xylanase (*orf_3772*), which is responsible mainly for attacking the xylan backbone and is similar to *Clostridium stercorarium* Xyn10B and *Thermotoga maritima* Xyn10A (Lee & Lee, 2014). By contrast, endo-1,4- β-xylanase (*orf_2117*) had no CBM domain, indicating that other substrate-binding regions might replace the function of CBM. The detection of endoglucanase, exoglucanase, and xylanase activities is in agreement with the presence of the aforementioned genes. It is interesting to note that the strain MP1 cultivated in LB medium containing 0.5% CMC had the lowest endoglucanase but activity of both enzymes increased at least 5.7 fold in TM3 medium supplemented with 2% rice bran, a low-cost agro-residue (Table S3). This result was considered as a promising strategy for reducing the cost of enzyme production and increasing enzyme efficiency.

Pectate trisaccharide-lyase (*orf_102*) and pectate lyase (*orf_1326*), which are involved in plant tissue maceration and modification of the cell wall structure (Atanasova et al., 2018), were identified based on the genomic analysis of strain MP1. Due to the ability to cleave pectin using a β-elimination mechanism, pectate lyases are produced either by bacteria living in close proximity with plants or by symbiotic gut bacteria (Biz et al., 2014; Jayani, Saxena & Gupta, 2005). Pectate lyases are important enzymes for industrial applications such as the beverage industry, pulp processing, waste treatment, leading to increasing attention of researchers over the world (Jayani, Saxena & Gupta, 2005; Zhao et al., 2018). Despite 72 genome sequences available at the time of writing and an array of extracellular enzymes found in *Cellulosimicrobium*, pectate lyases have not been identified yet*. C. cellulans* MP1 seemed to have acquired 2 different pectate lyases to establish itself in the termite gut. Additionally, pectinase produced by strain MP1 was low activity due to unoptimized medium. The production of pectinase can be optimized by using the response surface methodology as well as the recombinant enzyme, which is an interesting subject for further studies.

Interestingly, the presence of genes related to the levan exopolysaccharide in the genome of *C. cellulans* MP1 was predictable. Levan is one of the two main types of fructan biopolymers, produced from sucrose through the action of levansucrase (GH68) (Feng et al., 2015). Many studies showed that the GH68 is present in a number of genera, including *Gluconobacter, Gluconacetobacter, Komagataeibacter, Asaia, Neoasaia, Bacillus*, and *Kozakia* (Jakob et al., 2019); however, it has not been reported in the genus *Cellulosimicrobium*. Our finding revealed that the *sacC1-levB-sacC2-ls* operon is not conserved across well-studied bacteria and that the *ls* gene is encoded for active levansucrase, catalyzing the synthesis of higher-molecular-weight levan in presence of sucrose, which may serve as carbon storage for *C. cellulans* MP1. The molecules are hydrolyzed to L-FOs by levan-degrading enzymes including *sacC12* and *levB* (Fig. 7C). Some studies indicate that levanase LevB hydrolyzes levan to generate levanbiose predominantly and SacC is reported to be active against levan and inulin leading to the formation of free fructose (Débarbouillé et al., 1991; Raga-Carbajal et al., 2018). In *B. subtilis*, anti-terminator, SacY is phosphorylated in presence of the excess sucrose, resulting in upregulation of conserved levansucrase *sacB* and two endolevanase *yveAB* (Pereira, Petit-Glatron & Chambert, 2001). Another study demonstrated that activation of *sacB* also is regulated by pleiotropic regulatory genes *degS/degU, degQ* and *degR* (Débarbouillé et al., 1991). While no apparent homologs of SacY have been identified in the chromosome of strain MP1, 4 transcriptional factors *degA* (*orf_114*, *orf_207, orf_1005, orf_2448*) and *degU* (*orf_296*, *orf_527*, *orf_1182, orf_2409*) are present. Known that deletion of *sacC* in *Zymomonas mobilis* enhanced levan production 15.5 g/L to 21.2 g/L (Senthilkumar et al., 2004), further investigation on the regulation mechanisms of this operon is an interesting subject for improving levan production.

Interestingly, Ls shares the 2 conserved cysteines (at positions 383 and 439) and an overall sequence identity of 57–62% with orthologs of gram-negative bacteria such as *Microbacterium saccharophilum, Burkholderia vietnamiensis, Gluconacetobacter diazotrophicus* (Fig. S2). This result was in contrast to most gram-positive levansucrases that lack a pair of conserved cysteine residues (Jakob et al., 2019). In *G. diazotrophicus* levansucrase, Cys339-Cys395 intramolecular disulfide bond plays an important role in protein folding and stability. Serin substitution for either Cys339 or Cys395 abolished sucrose hydrolysis activity and levan-forming activity via preventing the extended loop between β-strands IIIB and IIIC with the insertion located between blades III and IV (Martínez-Fleites et al., 2005). Further crystal structure analysis is required to reveal the conformational changes of *C. cellulans* Ls upon reduction and oxidation conditions.

## Conclusions

This study highlighted the ability of strain MP1 to degrade cellulose and hemicellulose and produce levan. Out of 65 isolated termite gut symbiotic bacteria, isolate MP1, identified as *C. cellulans,* exhibited the highest specific cellulase activity. The genome of *C. cellulans* MP1 is one of 17 genomes of *C. cellulans* that are released onto the NCBI genome database, but it is the first sequence that has been reported in detail from a biotechnological perspective. Both genomic and experimental evidence proved that *C. cellulans* MP1 possesses 30 cellulose and 21 hemicellulose-related sequences, which were functionally redundant for endoglucanases, endoxylanase, β-glucosidases, xylanase, β-xylosidases, arabinofuranosidase, and pectate lyase. Moreover, the *sacC1-levB-sacC2-ls* operon involved in levan and L-FOs production was pronounced for the first time, which could be a selective advantage during host-adaptation and colonization. These findings not only enrich the genome database but also provide a valuable source of information to continue research into the potential applications of *C. cellulans* MP1, including its possible use in the biofuel, pulp and paper, and pharmaceutical industries.

##  Supplemental Information

10.7717/peerj.11839/supp-1 Supplemental Information 1 Endoglucanase and xylanase of strain MP1 cultivation at interval times

10.7717/peerj.11839/supp-2 Supplemental Information 2 Time course of levan production by the strain MP1

10.7717/peerj.11839/supp-3 Supplemental Information 3 CMCase and FPase activities of strain MP1 at interval times of cultivation

10.7717/peerj.11839/supp-4 Supplemental Information 4 Accession numbers of the 16S rDNA gene and genomic sequences of the strain MP1

10.7717/peerj.11839/supp-5 Supplemental Information 5 CMCase and FPase activities of strain MP1 with cultivation times

10.7717/peerj.11839/supp-6 Supplemental Information 6 Physiological and biochemical characteristics of strain** MP1

10.7717/peerj.11839/supp-7 Supplemental Information 7 Predicted genes associated cellulose and hemicellulose degradation

10.7717/peerj.11839/supp-8 Supplemental Information 8 Multiple genome alignments were generated by mapping genome sequences of three closely related *C. cellulans* strains against MP1

10.7717/peerj.11839/supp-9 Supplemental Information 9 Protein sequence alignments of levansucrase homologs across known bacteria Alignment of Ls homologs was performed with Clustal W. Enzyme source and Protein IDs correspond to: Gdia, *Gluconacetobacter diazotrophicus* (WP_012222901); Bvie, *Burkholderia vietnamiensis* (WP_011882121); Mce, *Cellulosimicrobium cellulans* MP1; Msac, *Microbacterium saccharophilum* (WP_147051238); Achr, *Azotobacter chroococcum* (WP_052264013). The red and blue color letters indicate percentage of amino acid identity. The conserved Cys is enclosed in the black box and marked with an asterisk (*).

## Additional Information and Declarations

### Data Availability

The following information was supplied regarding data availability: The raw data is available in the Supplemental Files. The 16S rDNA gene (MW534740) and genomic sequences (JAFGYF000000000) of the strain *Cellulosimicrobium cellulans* MP1 are available at NCBI GenBank.
