# Supplementary material for: A genomic perspective on the potential of termite-associated Cellulosimicrobium cellulans MP1 as producer of plant biomass-acting enzymes and exopolysaccharides
Source: PeerJ. 2021 Jul 28;9:e11839. doi: 10.7717/peerj.11839 (PMC8325422; doi:10.7717/peerj.11839)
Supplement: Supplemental Information 4 [file peerj-09-11839-s004.docx]

The 16S rDNA gene and genomic sequences of the strain *Cellulosimicrobium cellulans* MP1 in the present study were deposited onto GenBank (NCBI) and released to public under accession numbers MW534740 and JAFGYF000000000, respectively.

Links:

<https://www.ncbi.nlm.nih.gov/nuccore/MW534740.1>

<https://www.ncbi.nlm.nih.gov/nuccore/JAFGYF000000000>

Information for the 16S rDNA gene sequence

# Cellulosimicrobium cellulans strain MP1 16S ribosomal RNA gene, partial sequence

GenBank: MW534740.1

[FASTA](https://www.ncbi.nlm.nih.gov/nuccore/MW534740.1?report=fasta) [Graphics](https://www.ncbi.nlm.nih.gov/nuccore/MW534740.1?report=graph)

[Go to:](https://www.ncbi.nlm.nih.gov/nuccore/MW534740.1#goto1966687909_0)

LOCUS MW534740 1510 bp DNA linear BCT 02-FEB-2021

DEFINITION Cellulosimicrobium cellulans strain MP1 16S ribosomal RNA gene,

partial sequence.

ACCESSION MW534740

VERSION MW534740.1

KEYWORDS .

SOURCE Cellulosimicrobium cellulans

ORGANISM [Cellulosimicrobium cellulans](https://www.ncbi.nlm.nih.gov/Taxonomy/Browser/wwwtax.cgi?id=1710)

Bacteria; Actinobacteria; Micrococcales; Promicromonosporaceae;

Cellulosimicrobium.

REFERENCE 1 (bases 1 to 1510)

AUTHORS Nguyen,V.T., Xuan,D.T., Ha,L.T., Tung,Q.N. and Tien,P.Q.

TITLE Genome mining of the cellulolytic Cellulosimicrobium cellulans MP1

isolated from wood-feeding termite

JOURNAL Unpublished

REFERENCE 2 (bases 1 to 1510)

AUTHORS Nguyen,V.T., Xuan,D.T., Ha,L.T., Tung,Q.N. and Tien,P.Q.

TITLE Direct Submission

JOURNAL Submitted (28-JAN-2021) Fermentation technology, Institute of

biotechnology, 18 Hoang Quoc Viet, Cau Giay, Ha Noi, Ha Noi, Ha Noi

100000, Viet Nam

COMMENT ##Assembly-Data-START##

Sequencing Technology :: ABI PRISM3100-Avant Genetic Analyzer

##Assembly-Data-END##

FEATURES Location/Qualifiers

source 1..1510

/organism="Cellulosimicrobium cellulans"

/mol_type="genomic DNA"

/strain="MP1"

/isolation_source="wood-feeding termite"

/host="wood-feeding termite"

/db_xref="taxon:[1710](https://www.ncbi.nlm.nih.gov/Taxonomy/Browser/wwwtax.cgi?id=1710)"

/country="Viet Nam"

[rRNA](https://www.ncbi.nlm.nih.gov/nuccore/MW534740.1?from=1&to=1510) <1..>1510

/product="16S ribosomal RNA"

ORIGIN

1 agagtttgat cctggctcag gacgaacgct ggcggcgtgc ttaacacatg caagtcgaac

61 gatgaagccc agcttgctgg gtggattagt ggcgaacggg tgagtaacac gtgagtaacc

121 tgcccttgac ttcgggataa ctccgggaaa ccggggctaa taccggatat gagccgtcct

181 cgcatggggg tggttggaaa gtttttcggt cagggatggg ctcgcggcct atcagcttgt

241 tggtggggtg atggcctacc aaggcgacga cgggtagccg gcctgagagg gcgaccggcc

301 acactgggac tgagacacgg cccagactcc tacgggaggc agcagtgggg aatattgcac

361 aatgggcgca agcctgatgc agcgacgccg cgtgagggat gaaggccttc gggttgtaaa

421 cctctttcag cagggaagaa gcgcaagtga cggtacctgc agaagaagcg ccggctaact

481 acgtgccagc agccgcggta atacgtaggg cgcaagcgtt gtccggaatt attgggcgta

541 aagagctcgt aggcggtttg tcgcgtctgg tgtgaaaact cgaggctcaa cctcgagctt

601 gcatcgggta cgggcagact agagtgcggt aggggagact ggaattcctg gtgtagcggt

661 ggaatgcgca gatatcagga ggaacaccga tggcgaaggc aggtctctgg gccgcaactg

721 acgctgagga gcgaaagcat ggggagcgaa caggattaga taccctggta gtccatgccg

781 taaacgttgg gcactaggtg tggggctcat tccacgagtt ccgtgccgca gcaaacgcat

841 taagtgcccc gcctggggag tacggccgca aggctaaaac tcaaaggaat tgacgggggc

901 ccgcacaagc ggcggagcat gcggattaat tcgatgcaac gcgaagaacc ttaccaaggc

961 ttgacatgca cgggaagccg ccagagatgg tggtctcttt ggacactcgt gcacaggtgg

1021 tgcatggttg tcgtcagctc gtgtcgtgag atgttgggtt aagtcccgca acgagcgcaa

1081 ccctcgtccc atgttgccag cgggttatgc cggggactca tgggagactg ccggggtcaa

1141 ctcggaggaa ggtggggatg acgtcaaatc atcatgcccc ttatgtcttg ggcttcacgc

1201 atgctacaat ggccggtaca aagggctgcg ataccgtaag gtggagcgaa tcccaaaaag

1261 ccggtctcag ttcggattgg ggtctgcaac tcgaccccat gaagtcggag tcgctagtaa

1321 tcgcagatca gcaacgctgc ggtgaatacg ttcccgggcc ttgtacacac cgcccgtcaa

1381 gtcacgaaag tcggtaacac ccgaagccca tggcccaacc gttcgcgggg ggagtggtcg

1441 aaggtgggac tggcgattgg gactaagtcg taacaaggta gccgtaccgg aaggtgcggc

1501 tggatcacct

//

Genomic information of the strain MP1

# Cellulosimicrobium cellulans strain MP1, whole genome shotgun sequencing project

GenBank: JAFGYF000000000.1

- This entry is the master record for a whole genome shotgun sequencing project and contains no sequence data.

[Go to:](https://www.ncbi.nlm.nih.gov/nuccore/JAFGYF000000000#goto1993247178_0)

LOCUS JAFGYF010000000 23 rc DNA linear BCT 23-FEB-2021

DEFINITION Cellulosimicrobium cellulans strain MP1, whole genome shotgun

sequencing project.

ACCESSION JAFGYF000000000

VERSION JAFGYF000000000.1

DBLINK BioProject: [PRJNA695363](https://www.ncbi.nlm.nih.gov/bioproject/PRJNA695363)

BioSample: [SAMN17612315](https://www.ncbi.nlm.nih.gov/biosample/SAMN17612315)

KEYWORDS WGS.

SOURCE Cellulosimicrobium cellulans

ORGANISM [Cellulosimicrobium cellulans](https://www.ncbi.nlm.nih.gov/Taxonomy/Browser/wwwtax.cgi?id=1710)

Bacteria; Actinobacteria; Micrococcales; Promicromonosporaceae;

Cellulosimicrobium.

REFERENCE 1 (bases 1 to 23)

AUTHORS Dao,T.-T.-X., Vu,T.-H.-N., Le,T.-H., Quach,N.-T., Le,P.-C. and

Phi,Q.-T.

TITLE A genomic perspective on the potential of symbiotic

Cellulosimicrobium cellulans MP1 as producer of plant

biomass-acting enzyme and exopolysaccharide

JOURNAL Unpublished

REFERENCE 2 (bases 1 to 23)

AUTHORS Dao,T.-T.-X., Vu,T.-H.-N., Le,T.-H., Quach,N.-T., Le,P.-C. and

Phi,Q.-T.

TITLE Direct Submission

JOURNAL Submitted (03-FEB-2021) Vietnam Academy of Science and Technology

(VAST) Culture Collection of Microorganisms, Institute of

Biotechnology, 18 Hoang Quoc Viet, Hanoi, Vietnam, Hanoi 100000,

Viet Nam

COMMENT The Cellulosimicrobium cellulans whole genome shotgun (WGS) project

has the project accession JAFGYF000000000. This version of the

project (01) has the accession number JAFGYF010000000, and consists

of sequences JAFGYF010000001-JAFGYF010000023.

The annotation was added by the NCBI Prokaryotic Genome Annotation

Pipeline (PGAP). Information about PGAP can be found here:

<https://www.ncbi.nlm.nih.gov/genome/annotation_prok/>

Bacteria and source DNA available from Quyet-Tien Phi.

##Genome-Assembly-Data-START##

Assembly Method :: SPAdes v. 3.13

Genome Representation :: Full

Expected Final Version :: Yes

Genome Coverage :: 75.96x

Sequencing Technology :: Illumina MiSeq

##Genome-Assembly-Data-END##

##Genome-Annotation-Data-START##

Annotation Provider :: NCBI

Annotation Date :: 02/18/2021 18:13:57

Annotation Pipeline :: NCBI Prokaryotic Genome

Annotation Pipeline (PGAP)

Annotation Method :: Best-placed reference protein

set; GeneMarkS-2+

Annotation Software revision :: 5.1

Features Annotated :: Gene; CDS; rRNA; tRNA; ncRNA;

repeat_region

Genes (total) :: 4,088

CDSs (total) :: 4,027

Genes (coding) :: 3,964

CDSs (with protein) :: 3,964

Genes (RNA) :: 61

rRNAs :: 1, 1, 1 (5S, 16S, 23S)

complete rRNAs :: 1, 1, 1 (5S, 16S, 23S)

tRNAs :: 55

ncRNAs :: 3

Pseudo Genes (total) :: 63

CDSs (without protein) :: 63

Pseudo Genes (ambiguous residues) :: 0 of 63

Pseudo Genes (frameshifted) :: 19 of 63

Pseudo Genes (incomplete) :: 44 of 63

Pseudo Genes (internal stop) :: 5 of 63

Pseudo Genes (multiple problems) :: 5 of 63

##Genome-Annotation-Data-END##

FEATURES Location/Qualifiers

source 1..23

/organism="Cellulosimicrobium cellulans"

/mol_type="genomic DNA"

/strain="MP1"

/isolation_source="gut"

/host="termite"

/db_xref="taxon:[1710](https://www.ncbi.nlm.nih.gov/Taxonomy/Browser/wwwtax.cgi?id=1710)"

/country="Viet Nam: Nghe An province"

/collection_date="2017"

WGS [JAFGYF010000001-JAFGYF010000023](https://www.ncbi.nlm.nih.gov/Traces/wgs/JAFGYF01?display=contigs)

//
