# Supplementary material for: A genomic perspective on the potential of termite-associated Cellulosimicrobium cellulans MP1 as producer of plant biomass-acting enzymes and exopolysaccharides
Source: PeerJ. 2021 Jul 28;9:e11839. doi: 10.7717/peerj.11839 (PMC8325422; doi:10.7717/peerj.11839)
Supplement: Supplemental Information 5 [file peerj-09-11839-s005.docx]

**Table S1:** **CMCase and FPase activities of strain MP1** **with cultivation times**

| **Time (h)** | **CMCase (IU/ml)** | **FPase (FPU/ml)** |
| --- | --- | --- |
| 24 h | 0.11 ± 0.05 | 0.06 ± 0.02 |
| 48 h | 0.66 ± 0.15 | 0.14 ± 0.05 |
| 72 h | 0.56 ± 0.10 | 0.33 ± 0.10 |
| 96 h | 0.53 ± 0.10 | 0.22 ± 0.10 |
