# Supplementary material for: A genomic perspective on the potential of termite-associated Cellulosimicrobium cellulans MP1 as producer of plant biomass-acting enzymes and exopolysaccharides
Source: PeerJ. 2021 Jul 28;9:e11839. doi: 10.7717/peerj.11839 (PMC8325422; doi:10.7717/peerj.11839)
Supplement: Supplemental Information 6 [file peerj-09-11839-s006.docx]

**Table S2: Physiological and biochemical characteristics of strain MP1**

| **Characteristic** | **Result** | **Characteristic** | **Result** |
| --- | --- | --- | --- |
| **Morphological characteristics** | | H_2_S production | - |
| Shape | Short rods | Nitrate reduction | + |
| Size | 0.4-0.7 × 1.3-2.7 | Voges Proskauer | - |
| Motility | - | β-Galactosidase | + |
| Gram staining | + | Arginine dihydrolase | - |
| Spore formation | - | Lactose | - |
| **Physiological properties** | | Lysine decarboxylase | - |
| Temperature range for growth | 25-37 ^o^C | Ornithine decarboxylase | - |
| Optimum temperature | 30 ^o^C | L-Arabinose | - |
| pH range for growth | 7-12 | Tryptophanedeaminase | - |
| Optimum pH | 10 | Amygdalin | + |
| NaCl range for growth | 1-4 % | D-Glucose | - |
| Optimum NaCl | 2% | Amygdalin | + |
| **Biochemical properties** | | D-Mannitol | - |
| Catalase | + | Inositol | - |
| Oxidase | - | D-Sorbitol | - |
| Amylase | + | D-arabitol | - |
| Indole formation | - | D-Sucrose | + |
| Cellulose | + | D-Melibiose | - |
| Gelatinase | - | Amygdalin | + |
| Urease | - | D-Raffinose |  |
