# Supplementary material for: A genomic perspective on the potential of termite-associated Cellulosimicrobium cellulans MP1 as producer of plant biomass-acting enzymes and exopolysaccharides
Source: PeerJ. 2021 Jul 28;9:e11839. doi: 10.7717/peerj.11839 (PMC8325422; doi:10.7717/peerj.11839)
Supplement: Supplemental Information 7 [file peerj-09-11839-s007.docx]

**Table S3: Predicted genes associated cellulose and hemicellulose degradation.**

| **Classification** | **Locus tag** | **Start** | **End** | **Predicted function** | **Domains organization** | **EC_number** |
| --- | --- | --- | --- | --- | --- | --- |
| Cellulose-related | Orf_00454 | 523959 | 525308 | Endoglucanase | GH6+CBM2 | 3.2.1.4 |
|  | Orf_01616 | 22815 | 25427 | Endoglucanase | GH9 + CBM2 | 3.2.1.4 |
|  | Orf_02130 | 91343 | 93151 | Endoglucanase | GH9 | 3.2.1.4 |
|  | Orf_02755 | 91402 | 92763 | Endoglucanase | GH9 | 3.2.1.4 |
|  | Orf_03244 | 116610 | 118211 | Endoglucanase | GH6 + CBM2 | 3.2.1.4 |
|  | Orf_01607 | 7509 | 10229 | Exoglucanase | GH48 + CBM2 | 3.2.1.91 |
|  | Orf_01610 | 14411 | 16708 | Exoglucanase | GH6 | 3.2.1.91 |
|  | Orf_01611 | 16894 | 18285 | Exoglucanase | GH10 + CBM2 | 3.2.1.91 |
|  | Orf_02289 | 275036 | 276349 | Lichenase | GH16 | 3.2.1.73 |
|  | Orf_02294 | 280823 | 283051 | β-glucosidase | GH3 | 3.2.1.21 |
|  | Orf_02388 | 381171 | 382340 | β-glucosidase | GH1 | 3.2.1.21 |
|  | Orf_02464 | 70115 | 71650 | β-glucosidase | GH1 | 3.2.1.21 |
|  | Orf_02606 | 235024 | 236514 | β-glucosidase | GH1 | 3.2.1.21 |
|  | Orf_03385 | 94791 | 97361 | β-glucosidase | GH3 | 3.2.1.21 |
|  | Orf_00401 | 458558 | 460957 | β-glucosidase | GH3 | 3.2.1.21 |
|  | Orf_00403 | 461726 | 464680 | β-glucosidase | GH3 | 3.2.1.21 |
|  | Orf_00404 | 464931 | 467480 | β-glucosidase | GH3 | 3.2.1.21 |
|  | Orf_00922 | 1020956 | 1023193 | β-glucosidase | GH3 | 3.2.1.21 |
|  | Orf_02704 | 36873 | 38741 | Oligo-1,6-glucosidase | GH13 | 3.2.1.10 |
|  | Orf_02893 | 239128 | 240975 | Oligo-1,6-glucosidase | GH13 | 3.2.1.10 |
|  | Orf_02898 | 246386 | 248215 | Maltodextrin glucosidase | GH13 | 3.2.1.20 |
|  | Orf_00802 | 916961 | 918610 | Glucan endo-1,3-beta-glucosidase | GH64 | 3.2.1.39 |
|  | Orf_03703 | 70887 | 73010 | β-galactosidase | GH42 | 3.2.1.23 |
|  | Orf_02449 | 49706 | 51454 | Trehalose-6-phosphate hydrolase | GH13 | 3.2.1.93 |
|  | Orf_00102 | 132501 | 135185 | Pectate trisaccharide-lyase | GH13 | 4.2.2.22 |
|  | Orf_01326 | 347180 | 348832 | Pectate lyase | PL1 | 4.2.2.2 |
|  | Orf_00019 | 23426 | 24880 | Levanase | GH32 | 3.2.1.80 |
|  | Orf_00020 | 24974 | 26539 | Levanbiose-producing levanase | GH32 | 3.2.1.64 |
|  | Orf_00021 | 26649 | 28310 | Levanase | GH32 | 3.2.1.80 |
|  | Orf_00022 | 28398 | 30248 | Levansucrase | GH68 | 2.4.1.10 |
| Hemicellulose-related | Orf_00905 | 1000314 | 1001750 | Mannan endo-1,4-beta-mannosidase | CBM2 | 3.2.1.78 |
|  | Orf_03988 | 5510 | 8671 | Xylosidase/arabinosidase | GH43+CBM2 | 3.2.1.37 |
|  | Orf_04004 | 35586 | 40523 | Xylosidase/arabinosidase | GH43 | 3.2.1.55 |
|  | Orf_04003 | 32873 | 35287 | Arabinoxylan arabinofuranohydrolase | GH43+ CBM6 | 3.2.1.55 |
|  | Orf_00035 | 50051 | 52078 | *α*-xylosidase | GH31 | 3.2.1.177 |
|  | Orf_02605 | 232568 | 234865 | *α*-xylosidase | GH31 | 3.2.1.177 |
|  | Orf_00024 | 31857 | 35456 | *α*-L-arabinofuranosidase | GH43 | 3.2.1.55 |
|  | Orf_02083 | 29570 | 31750 | *α*-L-arabinofuranosidase | GH43 +CBM35 | 3.2.1.55 |
|  | Orf_03386 | 97358 | 98938 | *α*-L-arabinofuranosidase | GH43 | 3.2.1.55 |
|  | Orf_03999 | 21370 | 23106 | *α*-L-arabinofuranosidase | GH44 | 3.2.1.55 |
|  | Orf_04000 | 23103 | 25241 | *α*-L-arabinofuranosidase | GH45 | 3.2.1.55 |
|  | Orf_04002 | 29831 | 32725 | *α*-L-arabinofuranosidase | GH43+CBM4_9 | 3.2.1.55 |
|  | Orf_00034 | 48087 | 50054 | Non-reducing end beta-L-arabinofuranosidase | GH127 | 3.2.1.185 |
|  | Orf_03650 | 7408 | 9393 | Non-reducing end beta-L-arabinofuranosidase | GH127 | 3.2.1.185 |
|  | Orf_04001 | 25482 | 29660 | Non-reducing end beta-L-arabinofuranosidase | GH127 | 3.2.1.185 |
|  | Orf_03698 | 64526 | 65335 | Exo-*α*-(1->6)-L-arabinofuranosidase | GH51 | 3.2.1.- |
|  | Orf_01000 | 1103722 | 1105329 | Endo-1,4- β-xylanase | GH10+CBM2 | 3.2.1.8 |
|  | Orf_02117 | 74218 | 76707 | Endo-1,4- β-xylanase | GH10 | 3.2.1.8 |
|  | Orf_03772 | 53590 | 57684 | Endo-1,4- β-xylanase | GH10+CBM9 | 3.2.1.8 |
|  | Orf_02247 | 226234 | 227550 | *α*-galactosidase | GH4 | 3.2.1.22 |
|  | Orf_00266 | 314739 | 316913 | *α*-galactosidase | GH36 | 3.2.1.22 |
