# Supplementary figures and images for: A genomic perspective on the potential of termite-associated Cellulosimicrobium cellulans MP1 as producer of plant biomass-acting enzymes and exopolysaccharides
Source: PeerJ. 2021 Jul 28;9:e11839. doi: 10.7717/peerj.11839 (PMC8325422; doi:10.7717/peerj.11839)

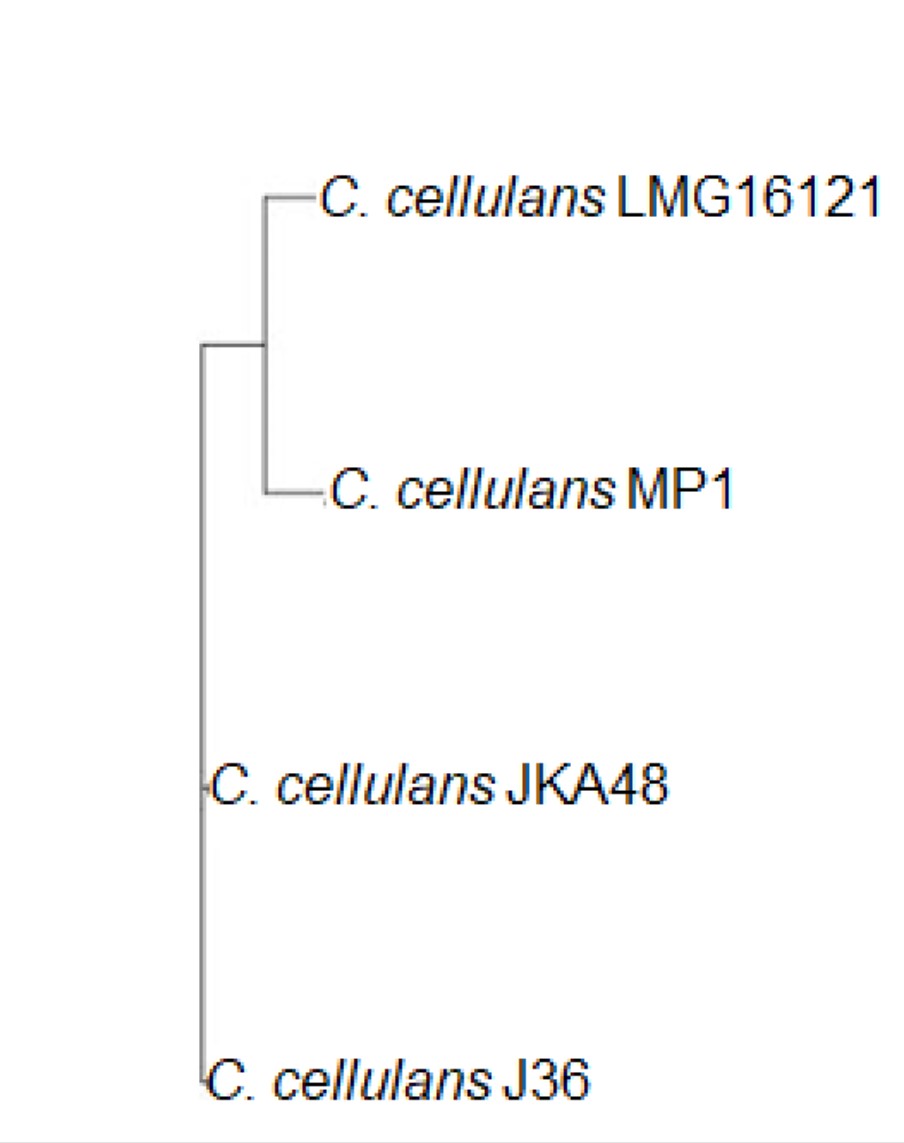

Supplement: Supplemental Information 8 [file peerj-09-11839-s008.jpg]

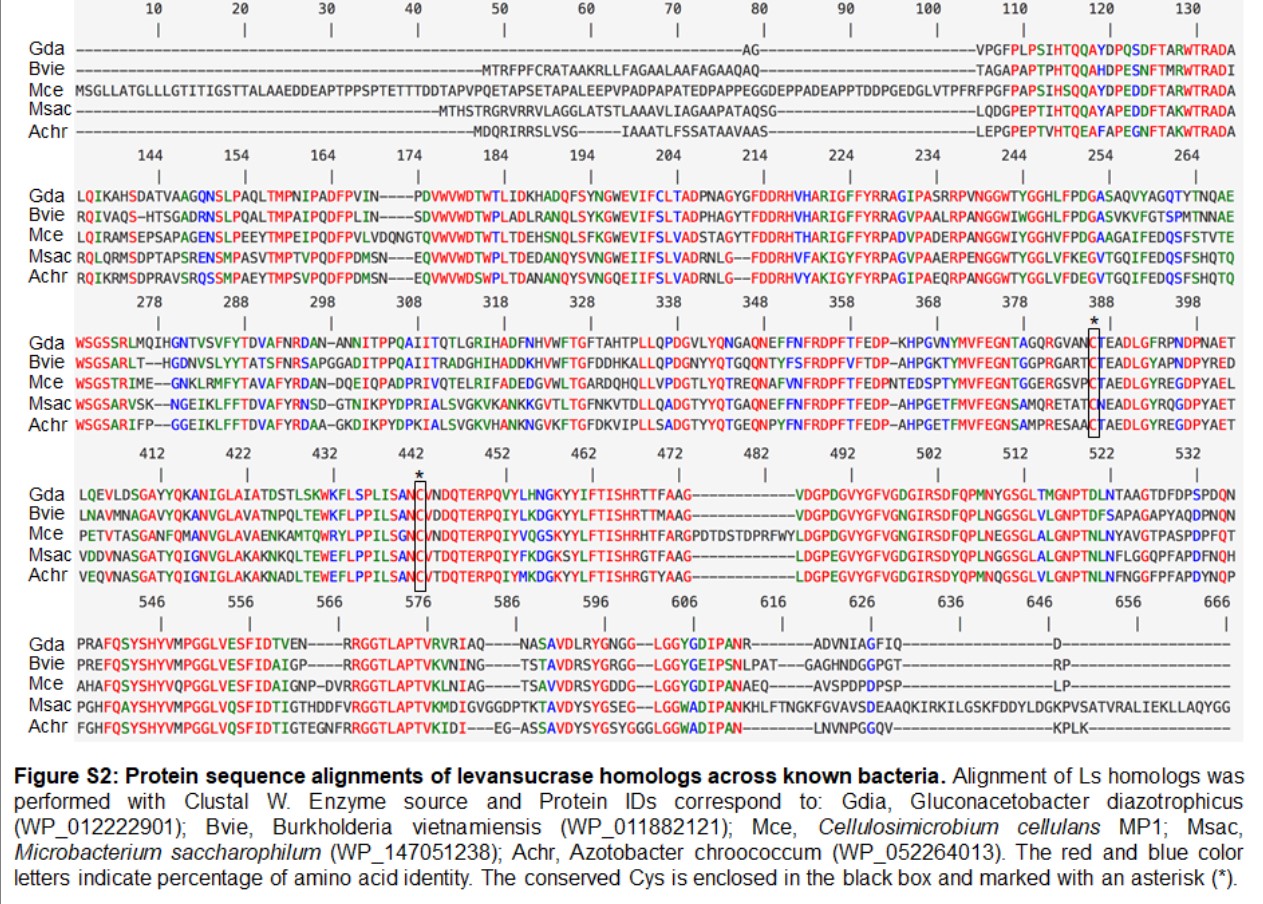

Supplement: Supplemental Information 9 — Alignment of Ls homologs was performed with Clustal W. Enzyme source and Protein IDs correspond to: Gdia, Gluconacetobacter diazotrophicus (WP_012222901); Bvie, Burkholderia vietnamiensis (WP_011882121); Mce, Cellulosimicrobium cellulans MP1; Msac, Microbacterium saccharophilum (WP_147051238); Achr, Azotobacter chroococcum (WP_052264013). The red and blue color letters indicate percentage of amino acid identity. The conserved Cys is enclosed in the black box and marked with an asterisk (*). [file peerj-09-11839-s009.jpg]
